# Structural Characteristics and Functional Implications of PM_2.5_ Bacterial Communities During Fall in Beijing and Shanghai, China

**DOI:** 10.3389/fmicb.2019.02369

**Published:** 2019-10-11

**Authors:** Yuanyuan Pan, Xianglong Pan, Hongwei Xiao, Huayun Xiao

**Affiliations:** Jiangxi Province Key Laboratory of the Causes and Control of Atmospheric Pollution, East China University of Technology, Nanchang, China

**Keywords:** PM_2.5_, airborne bacteria, bacterial structure, functions, environmental factors

## Abstract

Air pollution characterized by fine particulate matter (PM_2.5_) frequently has occurred in China, and has posed threats to human health. The physiochemical characteristics of airborne PM_2.5_ have been extensively studied, but its bacterial structures and functions have not yet been well studied. Herein, we focused on the structural characteristics and functional implications of airborne bacteria under different pollution levels in Beijing and Shanghai. The α- and β-diversities showed no obvious difference in two cities (*p* > 0.05). The dominant phyla Proteobacteria, Firmicutes, and Actinobacteria with total abundance of over 92% were found in all PM_2.5_ samples. The results of weighted unifrac non-metric multidimensional scaling (NMDS) suggested that air pollution was no obviously correlated with bacterial community but dispersed disorderly. Furthermore, canonical correlation analysis (CCA) and permutation test indicated that ${\text{NH}}_{4}^{+}$, ${\text{SO}}_{4}^{2-}$, and wind speed were the key factors that associated with airborne bacterial community structure. Chemical components of particulate matter played more important role in structuring bacterial community than meteorological conditions based on the result of partial CCA. In addition, the annotation of metabolic pathway suggested that the predominant genus *Pseudomonas* was obviously correlated with disease infections. Several dominant species might contribute to organic degradation, nitrogen cycles, and ice-nuclei activities in environments. Overall, this work enhanced our understanding of functions of airborne bacteria and highlighted their potential role in atmospheric chemical progresses.

## Introduction

Severe urban air pollution caused by particulate matters is a hot environmental issue in recent years due to its great potential influences on atmospheric and terrestrial ecosystems (Delort et al., 2010; Pandey et al., 2014). Specially, it induces human health risks such as cardiovascular diseases, respiratory infections, and lung cancer (Esposito et al., 2012; Pope and Dockery, 2013; Maclntyre et al., 2014). Previously, extensive studies have been carried out in studying chemical composition of particulate matter, which are considered to be the key factors of these harmful influences (Sun et al., 2013; Chen et al., 2017). Actually, the airborne microorganisms also might pose threat to human, animals, and plants. Recent studies showed that microorganisms were one of main components in the airborne particulate matters, occupying about 10% of total aerosol particles (Wei et al., 2015). Among these microorganisms, bacteria accounted for >80% (Cao et al., 2014), with the abundance from 10^5^ to 10^6^ cells/m^3^ in near-surface atmosphere (Bowers et al., 2011; Wei et al., 2016). For one thing, the sizes of bacterial and fungal cells were reported to be ranged from 1.1 to 2.1 μm and from 2.1 to 3.3 μm aerodynamic diameters, respectively (Raisi et al., 2013), easily penetrating and depositing into tracheobronchial and alveolar regions (Brook et al., 2004). For another thing, several airborne bacteria could be the sources of pathogens and allergens for human or plant (Woo et al., 2013; Cao et al., 2014; Du et al., 2018). In addition, studies found that bacteria in clouds also could act as cloud condensation nuclei or ice nuclei and then influence the cloud and rain formation (Bauer et al., 2003; Amato et al., 2015; Hara et al., 2016). Even some of these bacteria were viable and could metabolize ammonia oxidization (Gao et al., 2016) or biotransformation of organic compounds (Delort et al., 2010; Vaïtilingom et al., 2010, 2013; Maki et al., 2018). Therefore, besides chemical composition, it is essential to understand the bacterial characteristics of airborne particles.

In regard to the relationships between pollution levels and airborne bacteria, most existing studies focused on the impact of pollution on bacterial cell concentration. Although both positive (Wei et al., 2016; Li et al., 2018; Lu et al., 2018) and negative correlations (Gao et al., 2015; Xu et al., 2017) between pollution levels and airborne bacterial concentrations were observed, it confirmed that air pollution influenced the airborne bacterial concentration. Besides, Zhong et al. (2016) found that microbial metabolic activities in particulate matter in hazy days were higher than that in sunny days. However, limited studies have been conducted for studying shifts of airborne bacterial community under different air pollution levels (Wei et al., 2016, 2017; Li et al., 2018). The results were inconsistent. For instance, Wei et al. (2016) indicated that air pollution posed no effect on bacteria structure due to the stable sources during sampling period, while Wei et al. (2017) and Li et al. (2018) found the opposite results. Wei et al. (2017) also found that potential pathogens such as *Empedobacter brevis* and *Staphylococcus equorum* were enriched in the polluted cloud. Thus, in order to understand effects of airborne bacteria on human health and ecosystem, more studies about effects of air pollution on airborne bacteria should be conducted.

In previous studies, meteorological parameters (relative humidity, wind speed, temperature) and gaseous components (NO_2_, SO_2_, O_3_, CO) are often discussed as the main factors affecting the airborne bacterial diversity and structure (Woo et al., 2013; Lee et al., 2017; Xu et al., 2017). Nevertheless, airborne particulate matter included abundant organic matters, nitrate, and sulfate (Guo et al., 2014). Although Woo et al. (2013) showed the bioaerosol composition were determined by climate rather than airborne pollutants, Wei et al. (2017) found major ions were vital for shifting bacterial structures in clouds. It suggested that both climate and chemical components in PM_2.5_ might affect the bacteria structure.

Beijing and Shanghai are two of the largest cities in China, and have more than 10 million people. Beijing is a hot city for particle pollution study because it has suffered from frequent polluted events (Cao et al., 2014; Wei et al., 2016), for example, about one-third of the fall of 2017 with the concentration of PM_2.5_ > 75 μg/m^3^. Although the air pollution in Shanghai is relative mild, air pollution occasionally happened such as November 7–8, 2017 with an average PM_2.5_ concentration of 98.5 μg/m^3^. Furthermore, few studies were conducted to study airborne bacteria in Shanghai. In this study, PM_2.5_ samples were collected from these two megacities in polluted and unpolluted days in fall of 2017. The relation between air pollution and bacterial structure in atmospheric particulate matter was discussed; the dominant factors influencing airborne bacterial community were explored; and the key potential functions of these airborne bacteria were predicted.

## Materials and Methods

### Collection of PM_2.5_ Samples

PM_2.5_ samples were collected from the roof top of buildings at Chinese Research Academy of Environmental Sciences (Located in Beijing, 40.04° N, 116.41° E) and Shanghai Jiao Tong University (Located in Shanghai, 31.02° N, 121.43° E), respectively, where there were no major pollution sources nearby. Sampling was conducted by two high-volume samplers (KC-1000, Qingdao Laoshan Electric Inc., China), both equipped with PM_2.5_ fractionating inlets. Sampling was operated in fall (2017.09–2017.11) with different pollution levels. The detailed sampling days were listed in Table 1. Each sample was collected at a flow rate of 1.05 m^3^/min for 23.5 h (9:00 am to 9:00 am the next day) onto a Tissuquartz membrane filter (PALL Life Sciences, 2500QAT-UP, 8 in × 10 in). Blank samples were collected similar to the sampling methods except without instrument operation. Prior to sampling, the membrane filters were sterilized at 450°C for 4 h in a Muffle furnace. One part of the sample each day was used to extract water soluble ions, and another part of sample was stored at −80°C until genomic DNA extraction.

**TABLE 1:** Mean mass concentration of PM_2.5_ gas pollutants and meteorological parameters during sampling period.

**Sampling index**	**Sampling time**	**PM_2.5_ (μg/m^3^)**	**SO_2_ (μg/m^3^)**	**CO (mg/m^3^)**	**NO_2_ (μg/m^3^)**	**O_3_ (μg/m^3^)**	***T* (°C)**	**WS (m/s)**	**RH (%)**
BJ-1	2017.9.16−9.20	32.32 (36.6)	2.95 (0.75)	0.48 (0.30)	56.62 (8.55)	49.87 (17.23)	23.39 (0.87)	1.63 (0.20)	42.00 (10.77)
BJ-2	2017.10.28−11.4	47.35 (29.65)	5.89 (4.66)	0.88 (0.38)	59.65 (22.46)	27.41 (10.81)	8.88 (2.13)	2.06 (1.21)	49.32 (11.82)
BJ-3	2017.9.1/2/8/9	111.10 (33.66)	3.26 (0.93)	1.03 (0.24)	49.33 (26.36)	87.33 (50.90)	24.77 (2.10)	1.94 (0.33)	73.75 (3.59)
BJ-4	2017.10.25−10.27	146.09 (62.25)	13.06 (4.07)	1.95 (0.48)	82.93 (3.95)	27.78 (9.01)	12.23 (0.26)	0.92 (0.19)	84.21 (2.31)
BJ-5	2017.11.5−11.7	115.47 (33.11)	3.33 (1.40)	1.26 (0.26)	72.22 (15.51)	34.31 (9.97)	9.23 (1.89)	1.33 (0.59)	62.33 (17.39)
SH-1	2017.9.14−9.17	15.40 (2.14)	8.94 (1.59)	0.64 (0.06)	16.87 (4.16)	94.55 (14.97)	24.18 (0.73)	3.56 (0.88)	72.25 (6.43)
SH-2	2017.10.11−10.17	10.08 (3.38)	6.48 (2.01)	0.78 (0.08)	39.32 (6.70)	40.28 (8.95)	18.66 (0.71)	3.68 (0.78)	78.70 (10.74)
SH-3	2017.9.6/18/19/26	60.04 (8.15)	18.97 (4.41)	1.49 (0.14)	82.73 (20.78)	53.24 (26.41)	16.95 (1.65)	2.58 (1.28)	70.38 (7.67)
SH-4	2017.11.24−11.29	64.52 (16.89)	15.98 (3.11)	1.18 (0.19)	73.22 (19.13)	52.31 (17.30)	12.58 (1.65)	2.33 (1.19)	69.98 (9.94)
SH-5	2017.11.3/7/8	98.56 (2.34)	11.84 (2.60)	1.11 (0.16)	47.57 (4.90)	97.71 (24.94)	26.48 (1.23)	1.88 (0.31)	77.53 (10.13)
Sig._total_		^∗∗^		^∗∗^	*			*	
Sig._BJ–SH_		*	^∗∗^			*	*	^∗∗^	^∗∗^

**T*, temperature; WS, wind speed; RH, relative humility. Data in parentheses indicated standard deviation. Sig._*total*_ indicated the statistical difference between polluted and unpolluted days with ^∗^*p* < 0.05, ^∗∗^*p* < 0.01. Sig._*BJ–SH*_ indicated the statistical difference between Beijing and Shanghai.*

The environmental pollutant indexes (PM_2.5_, SO_2_, NO_2_, CO, and O_3_) and meteorological parameters (wind speed, temperature, and relative humility) per hour were obtained from the environmental monitoring stations at Beijing Olympic Sports Center and Shanghai Normal University, respectively, which were the nearest monitoring stations to sampling sites.

### Extraction and Measurement of Water-Soluble Ions

One-eighth of membrane filter (4 in × 2.5 in) per day was cut into a 50-mL centrifuge tube and then submerged with 50 mL deionized water. After ultrasonication extraction for 15 min at room temperature, the extract was filtered with 0.45 μm Teflon filter. Dionex Aquion (ICS-90, Thermo Scientific) were used to analyze the concentrations of water soluble cations (${\text{NH}}_{4}^{+}$, Na^+^, K^+^, Ca^2+^, and Mg^2+^), anions (${\text{NO}}_{3}^{-}$, ${\text{SO}}_{4}^{2-}$, F^–^, and Cl^–^) and organic acids (HCOOH, CH_3_COOH, C_2_H_2_O_4_, C_4_H_6_O_4_, C_5_H_8_O_4_, CH_4_O_3_S) in the filtrate. The pH was detected with a precision pH meter (PHS-3C, Leici Instruments Co., Shanghai, China).

### DNA Extraction and Sequencing

Considering the low DNA yield of airborne sample, one-eighth aerosol samples (about 185 m^3^ air) from several days were combined as one sample for the DNA extraction according to the method of Du et al. (2018) (see Table 1 for details). After the membrane filters were fully ground to powder in liquid nitrogen, 0.5 g sample was weighed to extract the total bacterial genomic DNA using the E.Z.N.A. Soil DNA Kit (Omega Bio-Tek, Norcross, GA, United States) according to the manufacturer’s instructions. To check sampling contamination, DNA of blank samples was extracted as samples. The extracted DNA was PCR amplified. No target fragments were observed for blank samples.

The bacterial 16S rRNA genes of aerosol samples were amplified using the PCR primers 341F (CCTACGGGNGGCW GCAG) and 806R (GGACTACHVGGGTATCTAAT) targeting the V3–V4 region (95°C for 2 min, followed by 27 cycles at 98°C for 10 s, 62°C for 30 s, 68°C for 30 s, and a final extension at 68°C for 10 min), where the eight-base barcodes were randomly added to the upstream of the universal primer to distinguish the different samples. PCR reactions were performed: 50 μL mixture containing 5 μL of 10× KOD buffer, 5 μL of 2.5 mM dNTPs, 1.5 μL of each primer (5 μM), 1 μL of KOD polymerase, and 100 ng of template DNA. After purification and quantification control, the V3–V4 tag PCR products were pooled with the other samples in equimolar and sequenced using 300 bp paired-end model on an Illumina HiSeq2500 platform in the GENE *DENOVO* Co., Ltd. (Guangzhou, China).

### Sequence Data Analysis

After sequencing, raw data were filtered according to the following two rules: (1) removing reads containing >10% of unknown nucleotides (N) and (2) removing reads containing <80% of base with quality (*Q*-value > 20). The paired end clean reads were merged as raw tags by the FLASH software (v 1.2.11) (Magoč and Salzberg, 2011). Low quality fragments were filtered out using QIIME software (v 1.9.1). Chimera checking and removal was performed via UCHIME algorithm on mothur platform. The effective tags were clustered into OTUs of ≥97% similarity using UPARSE software (Edgar, 2013). Singletons were removed from the whole sequence data set. The α-diversities (Chao1, Simpson, Shannon, Ace, and goods_coverage) were calculated in QIIME (v 1.9.1). The dissimilarity test [non-metric multidimensional scaling (NMDS)] based on Bray–Curtis similarity distance matrices was performed by the Vegan package in R 3.4.1. To acquire bacterial community function, Phylogenetic Investigation of Communities by Reconstruction of Unobserved States (PICRUSt, v 1.0) was performed (Langille et al., 2013): firstly, predicting gene content for each OTU sequence based on Greengenes database; secondly, finding the reference genomes whose metabolic pathway has been annotated; and then summarizing their Kyoto Encyclopedia of Genes and Genomes (KEGG) pathways according to the OTU abundance. The dominant OTU sequences were also blasted in the NCBI database to find the potentially corresponding strains and found out their ecological functions according to previous reports. And the canonical correlation analysis (CCA) was used to demonstrate the correlation between bacterial community and environmental factors. Monte Carlo permutation test was used to verify the significance of environmental factors related to bacterial community. Furthermore, the partial CCA (pCCA) was performed to compare the contributions of nutrients and meteorological conditions on the airborne bacterial structure (Liu et al., 2013). Raw 16S rRNA gene sequences reported here were deposited into the NCBI Sequence Read Archive (SRA^1^) database (Accession Number: SRP167452).

## Results

### Definition and General Characteristics of Unpolluted and Polluted Days

**FIGURE 1:**
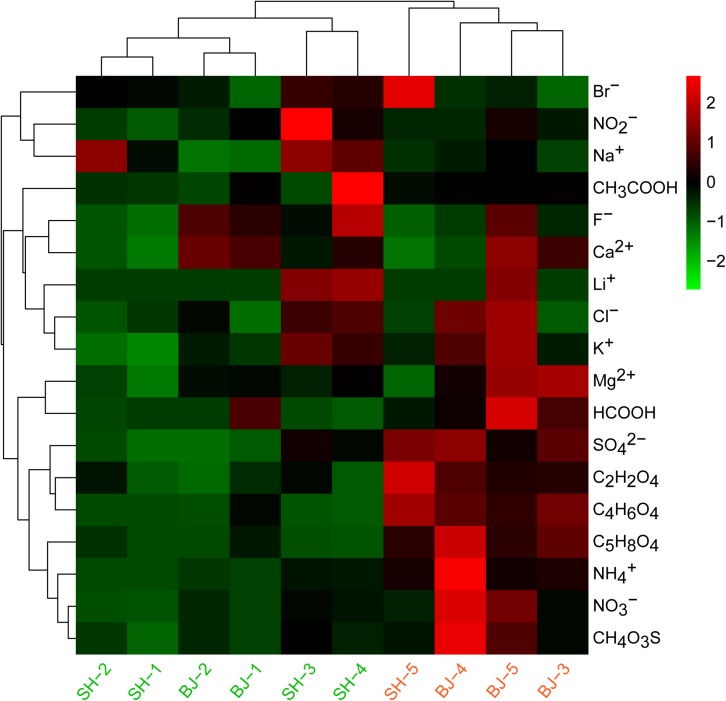
Heatmap based on concentrations of water soluble components in all PM_2.5_ samples. Red color indicated the higher concentration compared to other samples, while green color indicated the lower concentration compared to other samples. Cluster was also analyzed for all samples.

In the present study, aerosols were sampled from two metropolis Beijing and Shanghai during fall. First, referring to the criteria of China Meteorological Administration (CMA), we divided samples into two levels: unpolluted (0 < PM_2.5_ < 75 μg/m^3^) and polluted (PM_2.5_ > 75 μg/m^3^) groups (Table 1). Next, combined with the water soluble ions, the classification of PM_2.5_ samples was confirmed by cluster analysis in Figure 1. Therefore, we categorized samples SH-1, SH-2, SH-3, SH-4, BJ-1, and BJ-2 as unpolluted samples and samples SH-5, BJ-3, BJ-4, and BJ-5 as polluted samples.

Similar chemical components were observed for all samples, and ${\text{NH}}_{4}^{+}$, ${\text{NO}}_{3}^{-}$, and ${\text{SO}}_{4}^{2-}$ (SNA) were dominant in water soluble extraction. From the point of city, there was no obvious difference for the concentrations of these water soluble ions in two cities due to the large concentration differences within group. However, air pollution levels influenced their concentrations (Figure 1). The sum of SNA increased as the PM_2.5_ concentrations (*R* = 0.90, *p* < 0.01), which occupied 90.66 ± 0.03% of total measured water soluble matters in polluted days, significantly higher than that in unpolluted days (76.34 ± 0.07%). The concentrations of ${\text{NH}}_{4}^{+}$ (12.71 μg/m^3^), ${\text{NO}}_{3}^{-}$ (34.68 μg/m^3^), and ${\text{SO}}_{4}^{2-}$ (11.91 μg/m^3^) in polluted days were two to four times higher than that in unpolluted days (*p* < 0.01). Besides these three major ions, several organic acids were also found to be obviously enriched in polluted days such as C_2_H_2_O_4_, C_4_H_6_O_4_, C_5_H_8_O_4_, and CH_4_O_3_S (Figure 1). Compared with unpolluted days, motor vehicle exhaust like CO, NO_2_ in polluted days were obviously higher with 1.40 and 70.08 μg/m^3^, respectively (Table 1). Wind speed (1.71 m/s) in polluted days was lower than that (2.53 m/s) in unpolluted days.

### Overview of Bacterial Communities in PM_2.5_

More than 60,000 raw sequences were obtained for each sample, among which there were 80.71–85.62% effective sequences after quality control. According to Shannon rarefaction curve (Supplementary Figure S1), there were enough sequencing numbers for further analyses of airborne bacterial communities. These effective sequences resulted in 206–567 operational taxonomic units (OTUs) at 97% sequence identify cutoff (Table 2). *T*-test results showed that no obvious difference was observed for α-diversity between two cities (*p* > 0.05). Similarly, there was no significant difference for α-diversity between polluted days and unpolluted days (*p* > 0.05). Pearson correlation analysis also confirmed that PM_2.5_ concentration was no obviously correlated with α-diversity (Supplementary Table S1).

**TABLE 2:** Summary of sequence numbers and α-diversity of atmospheric bacterial community in PM_2.5_ obtained by 16S rRNA genes sequencing on the illumina Hiseq platforms.

**Samples**	**Raw Seqs^a^**	**Treated Seqs**	**OTUs**	**Shannon**	**Simpson**	**Chao1**	**Ace**	**Goods_coverage**
BJ-1	75514	64035	567	4.17	0.83	636.3	642.2	0.9984
BJ-2	69033	55714	502	5.49	0.94	589.3	576.8	0.9983
BJ-3	81092	68414	279	2.82	0.75	382.5	402.4	0.9986
BJ-4	80714	65290	343	3.91	0.88	439.9	474.9	0.9983
BJ-5	70216	57499	400	4.43	0.88	495.2	487.7	0.9984
SH-1	69821	58961	206	2.64	0.75	341.4	348.5	0.9985
SH-2	64908	54104	221	3.13	0.82	313.5	309.1	0.9986
SH-3	75630	63796	442	3.62	0.80	507.3	503.1	0.9986
SH-4	73288	61353	267	3.38	0.83	378.3	383.4	0.9985
SH-5	63172	54091	230	3.11	0.80	369.5	368.4	0.9983

*^*a*^Seqs means sequences.*

**FIGURE 2:**
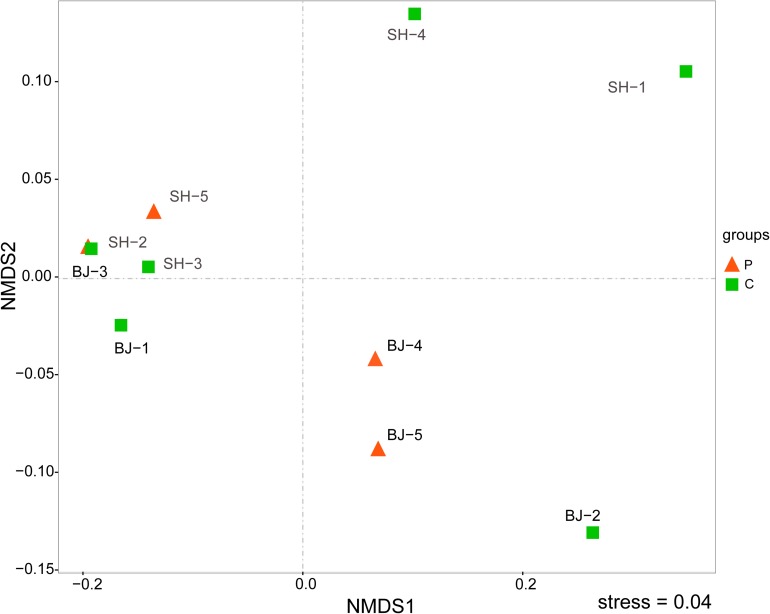
Comparing the bacterial communities in unpolluted and polluted samples using NMDS analysis at the OTU level. Red color represented polluted samples and green color referred unpolluted samples.

The NMDS result at OTU level further showed bacterial community structures of aerosol samples (Figure 2). The stress value was 0.04, which suggested the NMDS result was representative. As shown in Figure 2, all samples were not distributed by city or by pollution levels (Anosim, *p* > 0.05), but randomly dispersed and diverged from each other. It meant that bacterial compositions of aerosol samples were different, and other factors not pollution level influenced the bacterial community structure in PM_2.5_.

### Bacterial Structural Characteristics of PM_2.5_

**FIGURE 3:**
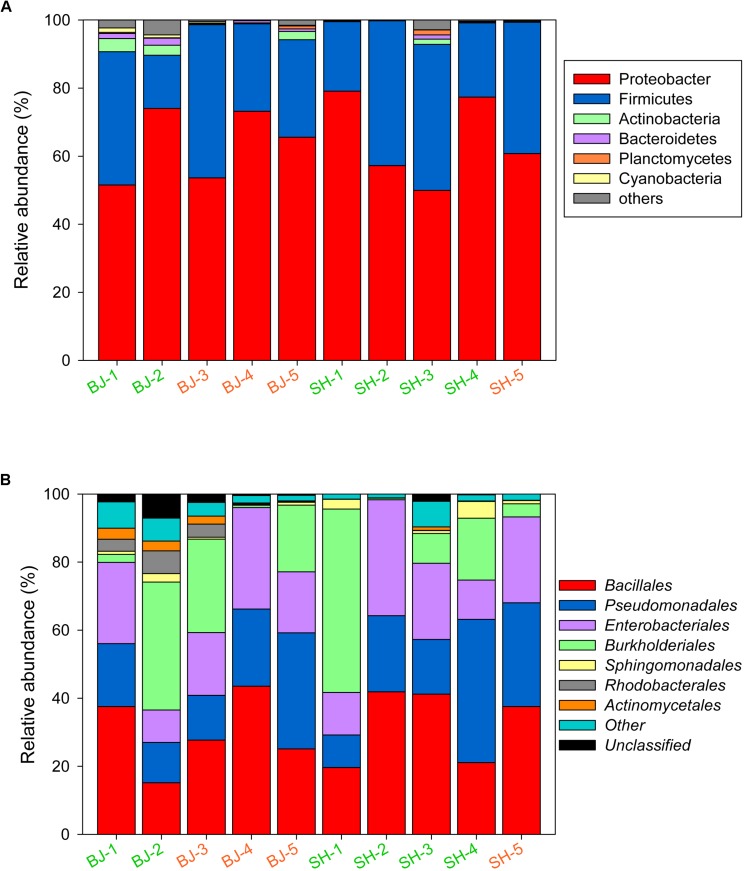
Bacterial community structure for PM_2.5_ samples at the phylum **(A)** and order level **(B)**. Taxonomic summary of the most abundant phylum (>1%) and order (>2%) was indicated in the figure.

As many as 19 bacterial phyla and 87 orders were identified in the PM_2.5_ samples. During the sampling time, the relative abundance of Proteobacteria in October and November in Beijing was higher than that in September, while Firmicutes was in contrast. No such trend was observed for Shanghai. The relative abundance of *Pseudomonadales* in Shanghai increased as the sampling time. However, based on the *t*-test results, no clear trend (*p* > 0.05) was observed for β-diversities of samples collected from Beijing and Shanghai, thus all samples were put together to analyze the bacterial compositions in polluted and unpolluted days. Among these phyla, Proteobacteria was the most abundant in all aerosol samples (Figure 3A), followed by Firmicutes and Actinobacteria, with total abundances of 92.61–99.87%. The phyla abundance of PM_2.5_ samples was not strongly correlated with pollution levels. The average abundance of Proteobacteria (63.30 ± 8.23%) in polluted days was close to that in unpolluted days (64.87 ± 13.42%). In contrast, the bacterial abundances varied greatly among samples. Firmicutes (39.16%) in BJ-1 was about 2.5 times that in BJ-2, although both belonged to unpolluted days (Figure 3A). Similar phenomenon was observed at the order level. The dominant orders of all samples included *Bacillales*, *Pseudomonadales*, *Enterobacteriales*, and *Burkholderales*, while the proportion of each order was distinct from sample to sample (Figure 3B). Pearson correlation analysis demonstrated that *Pseudomonadales* were positively correlated with ${\text{NH}}_{4}^{+}$ (*r* = 0.73, *p* < 0.05). Overall, the bacterial structure of each sample had its own characteristics, independent of air pollution levels (PM_2.5_ concentration), and environmental factors had obvious influences on airborne bacterial composition.

### Environmental Factors Shaping the Bacterial Community Structure

**FIGURE 4:**
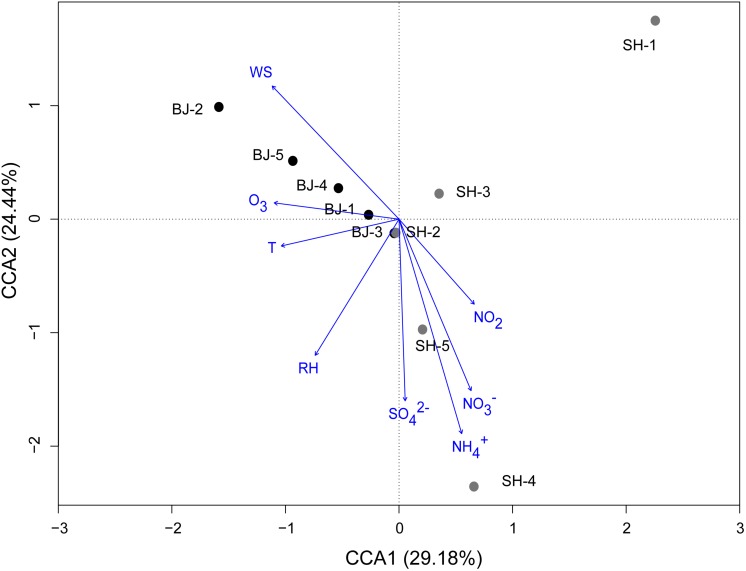
Canonical correspondence analysis (CCA) of bacterial community structures (OTUs) from 10 PM_2.5_ samples with respect to the eight environmental and meteorological variables. Arrows indicate the direction and magnitude of measurable variables associated with community structures.

In order to further confirm the impact of environmental and meteorological conditions on airborne bacterial community, CCA and pCCA were operated at the OTU level. CCA biplot showed that 96.90% of the total variance of airborne bacterial community, calculated from the ratio of constrained inertia and total inertia, was totally explained by the environmental and meteorological factors (Figure 4). Based on the result of Monte Carlo permutation test (*p* = 0.04), this explanation was significant. Among these, the first two components (CCA1 and CCA2) together explained 53.62% of the total variation of OTU structure (Figure 4). Meanwhile, ${\text{NH}}_{4}^{+}$, wind speed, and ${\text{SO}}_{4}^{2-}$ were the predominant factors which shifted the bacterial community structure (*r* = 0.758, 0.552, and 0.498, respectively; *p* < 0.05). The results suggested that both ions and meteorological conditions together contributed to the structure of airborne microbial community. The pCCA result further showed that the explanation of chemical ions (60.35%) for the bacterial shift was higher than that of meteorological factors (38.57%).

### Functional Estimation for PM_2.5_ Bacterial Communities

**FIGURE 5:**
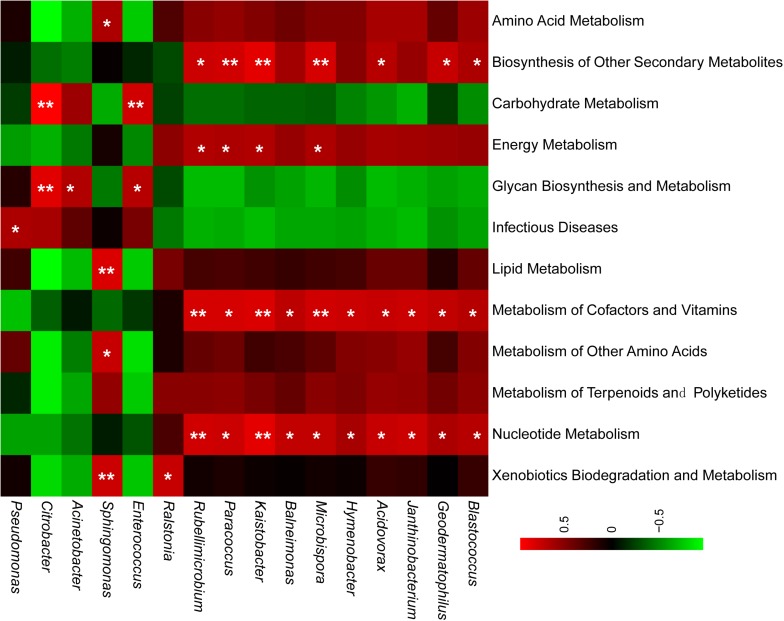
Bacterial taxa are related to KEGG pathways. Pearson’s correlation coefficients were calculated for the relative abundances of genera and KEGG pathways. Red color referred to the positive correlation and green color indicated a negative correlation. Significant correlation was marked with ^∗^*p* < 0.05, ^∗∗^*p* < 0.01.

Although the species and abundances of airborne microbes were gradually concerned, their functions were still not recognized, particularly their contribution for aerosol particle formation. PICRUSt software offered potential for gaining insight into the drivers of function dynamics during the formation process of particle pollution. In this study, weighted nearest sequenced taxon index (NSTI) of all samples were lower than 0.1, which suggested that the PICRUSt results were reliable. Overall, 232 reported KEGG pathways were obtained according to the annotated reference genomes for OTU sequences in PM_2.5_ samples, and the predominant pathways were mainly related to metabolism, environmental information processing, genetic information processing, cellular processes, and human diseases. This study focused on the pathways of bacterial metabolism and human diseases. Firstly, the proportions of these pathways in polluted days were similar with that in unpolluted days. Secondly, the airborne bacterial community contained a versatile group (Figure 5). For example, *Sphingomonas* and *Ralstonia* had a significant correlation with xenobiotics biodegradation and metabolism. *Pseudomonas* were positively associated with infection disease (Figure 5). About the abundant OTUs with relative abundance >10%, previous studies demonstrated that their related species participated in multiple ecological functions such as N-cycling, forming atmospheric ice nuclei, organic degradation, and opportunistic pathogenic (Table 3).

**TABLE 3:** The dominant bacteria (relative abundance >10%) in PM_2.5_ samples correlated with the potential ecological function.

**Potential species**	**Ecological role**	**References**
*Exiguobacterium aurantiacum*	Organic degradation	Jeswani and Mukherji, 2013
*Citrobacter freundii*	Nitrate reduction	Li et al., 2015
*Pseudomonas grimontii*	Denitrification	Cai et al., 2010
*Acinetobacter junii*	Opportunistic pathogens	Nemec et al., 2016
	Nitrification/denitrification	Ren et al., 2014
*Pseudomonas putida*	Organic degradation	Gomes et al., 2005
	Ice nuclei	Lee et al., 1995
*Pseudomonas protegens*	Plant protection	Ramette et al., 2011
*Ralstonia pickettii*	Organic degradation	Kahng et al., 2000
*Comamonas testosteroni*	Organic degradation	Hollender et al., 1997

## Discussion

Effects of airborne particulate matter on human health have been fully illustrated in terms of chemical composition (Esposito et al., 2012; Pope and Dockery, 2013; Maclntyre et al., 2014). Besides the harm of chemical components, airborne bacteria are ubiquitous (10^5^–10^6^ cells/m^3^) and also may cause respiratory diseases such as allergy and asthma (Brook et al., 2004; Woo et al., 2013). Although the number of concentration of airborne bacteria has been confirmed to be closely correlated with the air pollution levels (Li et al., 2015; Wei et al., 2016; Du et al., 2018; Lu et al., 2018), an understanding of the structural and functional response of airborne bacteria to air pollution is still elusive. In the present study, PM_2.5_ samples were collected to investigate the relationship of the structure and function implications of airborne bacterial community with air pollution.

Compared with unpolluted days, the concentrations of PM_2.5_ and chemical species generally increased in polluted days (Zhang et al., 2016). On the basis of this aspect, in this study, six samples were classified into unpolluted samples and four samples were classified into polluted samples (Table 1 and Figure 1). However, despite several OTUs belonging to *Acinetobacter* and *Massilia* enriched in polluted days, the airborne bacterial communities showed no significant correlation with air pollution. On the one hand, air pollution posed no impact on all α-diversity indices (Shannon index, Simpson index, Chao1, and Ace), and these parameters showed low correlation with PM_2.5_ concentration (*r* < 0. 2) (Supplementary Table S1). On the other hand, consistent with other studies about airborne bacteria in Beijing (Cao et al., 2014; Lee et al., 2017; Du et al., 2018; Li et al., 2018), the dominant bacteria in PM_2.5_ samples belonged to *Bacillales*, *Pseudomonadales*, *Enterobacteriales*, *Burkholderiales*, and *Sphingomonadales*, which were similar in whether polluted or unpolluted days (Figure 3). Notably, airborne bacterial structure was not obviously related to air pollution (Figure 2). The proportions of dominant bacteria varied from each other, even from the same city and pollution level (Figure 3). The large differences within the group lowered the differences between groups. It suggested that there were some factors contributing to these differences.

Generally, meteorological conditions such as relative humidity, wind speed, the concentrations of SO_2_ and NO_2_, were considered to influence the airborne bacterial concentration and structure (Cao et al., 2014; Wei et al., 2017; Xu et al., 2017; Li et al., 2018). High humidity favored the survival of microbes in the air (Cao et al., 2014). SO_2_ and NO_2_ inhibited the growth and breeding of airborne bacteria (Xu et al., 2017). Wind speed conducted the structure of bacterial community in cloud (Evans et al., 2006; Wei et al., 2017). In this study, high explanation (53.62% by CCA1 and CCA2) was obtained from combining meteorological parameters and water soluble compounds in PM_2.5_ (Figure 4). This explanation proportion was higher than the result (13.67%) of Li et al. (2018), while it was comparable with the result (65.9%) of Wei et al. (2017). In accordance with the results in this study, major water soluble ions and wind speed were the crucial environmental factor, different from Woo et al. (2013) who found climate not chemical compounds impacted airborne bacterial community. This might be due to the fact that soluble ions could act as the metabolic substrates. For example, Gao et al. (2016) confirmed the function of ammonia oxidation for the bacteria in PM_2.5_. N-cycling-related OTUs were also found in this study (Table 3). Overall, air pollution showed no obvious correlation with bacterial community (Anosim, *p* > 0.05) as it reflected the combined influence of chemical compounds and meteorological conditions. Nevertheless, ${\text{SO}}_{4}^{2-}$ and ${\text{NO}}_{3}^{-}$ different from other compounds played an important role in conducting bacterial community of PM_2.5_.

More to the point, part of airborne bacteria was viable (Hara and Zhang, 2012; Zhong et al., 2016; Li et al., 2018), but there was no systematic understanding about the functions of these bacteria. In the previous reports, pathogenic bacteria in air particulate matter were the focus of airborne bacterial function, and some pathogens has been identified in the air particulate matter such as *Pseudomonas syringae* and *Pseudomonas cichorii* (Pauwelyn et al., 2011; Vaïtilingom et al., 2013; Du et al., 2018). In the present study, the dominant genus *Pseudomonas* showed significant correlation with infectious diseases (Figure 5). *Acinetobacter modestus* (Nemec et al., 2016), an opportunistic pathogenic specie, was abundant in the PM_2.5_ samples (Table 3). Other common genera of potential human pathogens including *Streptococcus* (Lin et al., 2003) and *Prevotella* (Ibrahim et al., 2017) were also detected in this study (Supplementary Figure S2). Besides the health risk, airborne bacteria may also play an important role in atmospheric biochemistry. The related species of several predominant OTUs, such as *Exiguobacterium aurantiacum*, *Pseudomonas putida*, *Ralstonia pickettii*, and *Comamonas testosterone*, have been reported to contribute to biodegrading hydrocarbons (Jeswani and Mukherji, 2013), phenolics, aromatic compounds (Hollender et al., 1997; Kahng et al., 2000; Gomes et al., 2005; Table 3). Pearson correlation analysis further confirmed that genera *Ralstonia* and *Sphingomonas* were significantly correlated with xenobiotics biodegradation and metabolism (*p* < 0.05) (Figure 5). Similar in the cloud, some airborne bacteria also act as an efficient ice nuclei because they could induce ice nucleation at a warmer temperature than mineral dusts (Amato et al., 2015). For example, the abundance related specie in this study, *P. putida*, was an ice nucleation active specie and showed the ice nucleation activity at −3°C (Hill et al., 2014). All these functions implied that airborne bacteria might be involved in changing meteorology and climate.

## Conclusion

Airborne bacteria, including their structure response to different pollution levels, correlation with environmental factors, and potential role in atmospheric progresses, remain elusive. In the present study, airborne bacterial structure showed no significant correlation between polluted and unpolluted days, and between two cities in fall. The relationship of environmental factors and meteorological conditions with airborne bacterial community was explored, and found that ${\text{NH}}_{4}^{+}$, ${\text{SO}}_{4}^{2-}$, and wind speed significantly structured the bacterial community. Besides health risk from several potential pathogenic genera (*Pseudomonas*, *Streptococcus*, and *Prevotella*), some abundant species related to organic degradation and nitrification/denitrification may contribute to atmospheric biochemistry process. This knowledge helps for the comprehensive understanding of bacterial community biodiversity and their potential role in atmospheric progress. However, in the future study, more aerosol samples obtained through long term such as a whole year and high resolution sampling should be conducted to further disclose the correlation between airborne bacterial diversity and air quality. In addition, studies depending on the RNA technique and biochemical culture experiments need to be performed to confirm the functions in the atmosphere.

## Data Availability Statement

Raw 16S rRNA gene sequences reported here were deposited into the NCBI Sequence Read Archive (SRA, http://www.ncbi.nlm.nih.gov/sra) database (Accession Number: SRP167452).

## Author Contributions

YP designed the study, performed the experiments, analyzed the data, and wrote the manuscript. XP performed the experiments and analyzed the data. HoX designed the experiments. HuX revised the manuscript.

## Conflict of Interest

The authors declare that the research was conducted in the absence of any commercial or financial relationships that could be construed as a potential conflict of interest.

## Footnotes

1 http://www.ncbi.nlm.nih.gov/sra
