# Supplementary material for: Structural Characteristics and Functional Implications of PM2.5 Bacterial Communities During Fall in Beijing and Shanghai, China
Source: Front Microbiol. 2019 Oct 11;10:2369. doi: 10.3389/fmicb.2019.02369 (PMC6798152; doi:10.3389/fmicb.2019.02369)
Supplement: Supplementary file 1 [file Data_Sheet_1.docx]

*Supplementary of*

**Structural characteristics and** **functional implications of** **PM_2.5_ bacterial communities during fall in Beijing and Shanghai, China**

**Yuanyuan Pan^1^, Xianglong Pan^1^, Hongwei Xiao^1^,** **Huayun Xiao^1*^**

^1^ Jiangxi Province Key Laboratory of the Causes and Control of Atmospheric Pollution, East China University of Technology, Nanchang, China

***Correspondence:**

Prof. Huayun Xiao

E-mail: [xiaohuayun@ecit.cn](mailto:xiaohuayun@ecit.cn)

**Fig. S1** Shannon index versus number of tags sampled for each PM_2.5_ sample.


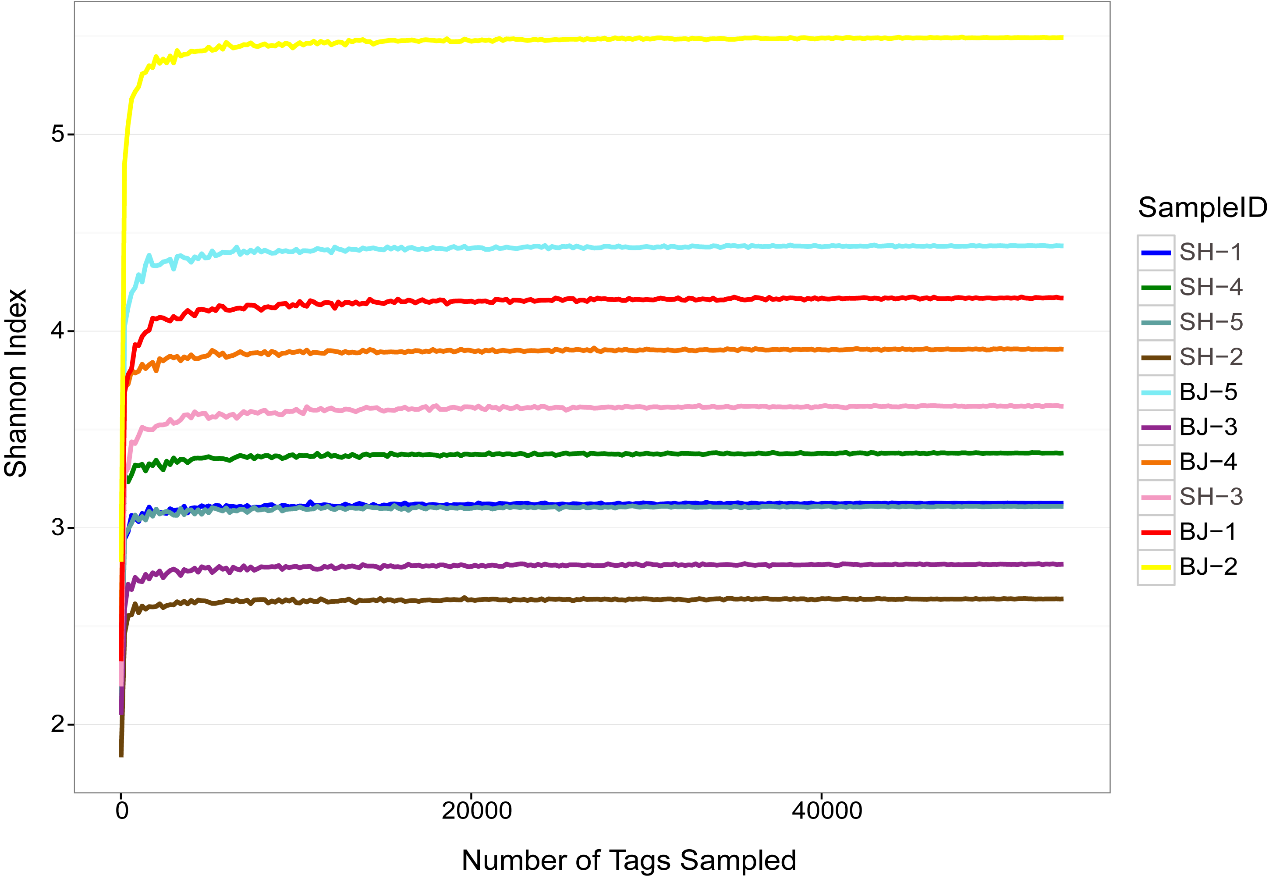


**Fig. S2** Heatmap at the genus level. Red color indicated the higher concentration compared to other samples, while green color indicated the lower concentration compared to other samples.


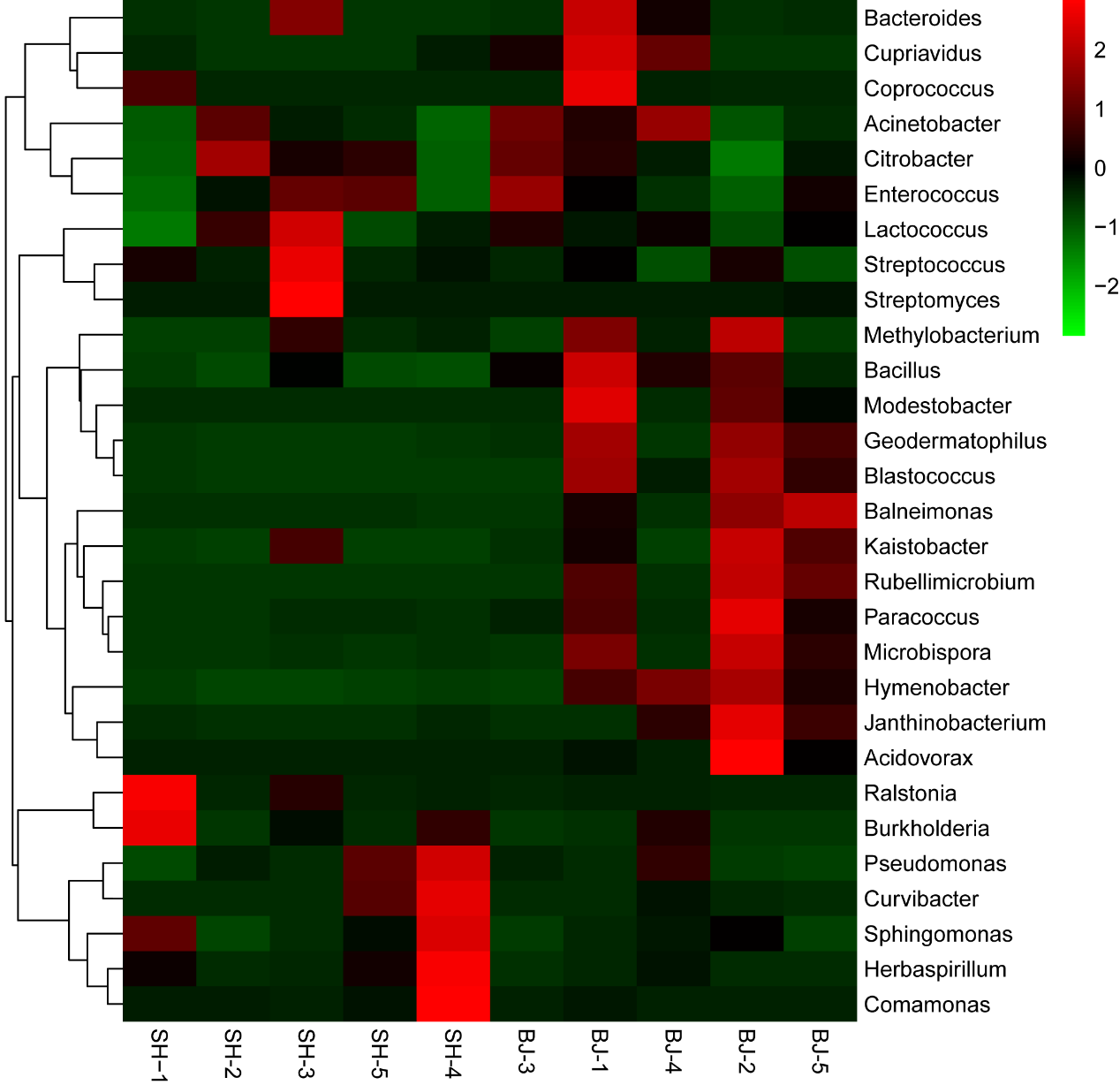


**Table S1** Pearson correlation coefficient between PM_2.5_ and α-diversity indices (p>0.05).

|  | OTU number | Shannon | Simpson | Chao1 | Ace |
| --- | --- | --- | --- | --- | --- |
| PM_2.5_ | -0.02 | 0.09 | 0.19 | 0.00 | 0.07 |
